# Cross-modal music integration in expert memory: Evidence from eye movements

**DOI:** 10.16910/jemr.11.2.4

**Published:** 2018-12-12

**Authors:** Véronique Drai-Zerbib, Thierry Baccino

**Affiliations:** LEAD, University of Bourgogne Dijon, France; CHART/LUTIN, University of Paris 8 Paris, France

**Keywords:** Expertise, music reading, eye tracking, cognition, cross-modal integration

## Abstract

The study investigated the cross-modal integration hypothesis for expert musicians using eye tracking. Twenty randomized excerpts of classical music were presented in two modes (auditory and visual), at the same time (simultaneously) or successively (sequentially). Musicians (N = 53, 26 experts and 27 non-experts) were asked to detect a note modified between the auditory and visual versions, either in the same major/minor key or violating the key. Experts carried out the task faster and with greater accuracy than non-experts. Sequential presentation was more difficult than simultaneous (longer fixations and higher error rates) and the modified notes were more easily detected when violating the key (fewer errors), but with longer fixations (speed/accuracy trade-off strategy). Experts detected the modified note faster, especially in the simultaneous condition in which cross-modal integration may be applied. These results support the hypothesis that the main difference between experts and non-experts derives from the difference in knowledge structures in memory built over time with practice. They also suggest that these high-level knowledge structures in memory contain harmony and tonal rules, arguing in favour of cross-modal integration capacities for experts, which are related to and can be explained by the long-term working memory (LTWM) model of expert memory (e.g. (18; 22).

## Introduction

Empirical studies observed a classical concert pianist reading to memorize *Clair de Lune* by Debussy in a few hours of training, using the musical structure of the score to organize her performance (9; 40). To achieve this level of expertise, musicians have to train in reading and performance over many years of learning.

Music reading consists of translating visuo-spatial symbols (notes and codification placed on the stave) into sounds. Expert reading is a fundamental competence in Western tonal musical practice. The code of Western music writing is an organized system of tones, called tonality, in which pitches and chords are hierarchically arranged. Thus, music notation is driven by rules and codifications governing tone and harmony using three types of information: sound pitch, time duration and aspects of musical performance, such as tempo, dynamics, phrasing and musical style (13).

What are the underlying cognitive processes of expert music reading? Reading a score is a highly demanding task that requires multimodal processing: perceptual (visual, auditory), cognitive (memory, planning) and motor (hand/voice execution). During this complex activity, the musician is able to extract the visual notation, identify and hierarchize from the score the composite musical elements (e.g. notes, rhythm, tonal and harmonic rules, dynamics, musical form…) and integrate them in a music situation model in order to play/sing in a virtuosic way. In the classical music tradition, an expert musician will be fluent in reading any piece of music. As in any activity of reading involving the oculomotor system, the eyes alternate short and rapid movements, called saccades, with brief periods of stationary time, called fixations (20; 20; 43 ). Visual information is extracted from short segments of text during fixations lasting about a quarter of second (for a review, see e.g. (62). Thus, eye tracking is an extremely helpful method for monitoring the direction (saccades), duration (fixations) and other properties of eye movements (amplitude, velocity, acceleration, pupil dilation, blinks, etc.) during cognitive activity (for a review, see (38). The duration and trajectory of the gaze depend on the task to be performed (78) and readers make longer fixations at points where processing loads are greater (eye-mind hypothesis; (44).

Since the early measurements taken by 20), eye-movement tracking has predominantly been used to investigate reading (60) and is increasingly being used to investigate music reading (for reviews, see (49; 61). In music reading, the underlying perceptual, cognitive and motor processes have been tracked through the gaze behaviour engaged in different reading processes, i.e. the visual recognition and pattern-matching paradigm (70), reading in the mind before playing (5), identifying a modification in the score (17, 18), sight-reading (concurrent reading and performing) (19; 32; 35; 57; 61; 77) and reading while conducting an orchestra (6)

Music reading studies conducted in eye-tracking research for Western tonal music have shown that expert musicians do not look at every note (72 ). The average fixation duration in music sight-reading is in the range of 200–400 ms (70). Mean fixation durations and dwell times are shorter and saccades are larger at a faster tempo and there are more fixations with more note symbols (26; 45). Adult novice readers gradually decrease the duration of their first pass fixations on scores over a training period of music sight-reading (57)

Research on eye movements has highlighted inter-individual differences related to musical expertise. Musicians have larger saccade amplitudes and shorter fixation durations when they are more skilled in music. Experts’ superior encoding capacities can be understood as related to: 1) an adaptation of oculomotor mechanisms (32; 61; 68); 2) the construction of an expert memory using an efficient process of pattern recognition (69; 70; 71) and the ability to access quickly a set of information in the long-term memory (22; 75); 3) cross-modal integrative music reading observed in most expert musicians (8; 18).

### Adaptation of Oculomotor Mechanisms

When considering the oculomotor perspective, expert musicians differ from non-expert musicians in terms of the number, duration and position of fixations on the musical stave. Expert musicians make fewer and shorter fixations (first pass and second pass fixations) and larger saccades than less-skilled musicians (5; 32; 61; 70) showing a relative independence from the written code on the score. Expert musicians also differ from non-experts in terms of their eye-hand or eye-voice span, as well as the distance to which the eyes scan the score ahead of the music performed by the hand or voice (68). Their gaze is slightly ahead of the movement execution compared to non-experts (41 ; 47; 68). Moreover, their eye-hand span is sensitive to the task requirements: pianists show a longer eye-hand span when playing at a slower tempo (1.3 s at a slow tempo vs. 0.7 s for a fast tempo; (1.3 s at a slow tempo vs. 0.7 s for a fast tempo;(26). Violinists show an eye span of around 1 s (2–6 notes), but are influenced by the characteristics of the notation and the structural complexity of the score (77).

### Music Reading and Expert Memory

How can reading differences between experts and non-experts musicians be explained? Models of expert memory may shed light on music reading and performance according to the level of expertise. Chunking models of expertise (11; 30; 71) are able to explain the perceptual structure processing involved in music reading through visual encoding (69) and retrieval (27). Expert musicians are able to recognize melodic, harmonic, or rhythmic patterns, resulting in chunks of notes that improve their sight-reading compared to novices. This chunking allows them to recognize the shape or profile of groups of notes rather than having to read all the notes written on the score. Thus, experts have an efficient pattern recognition process that they can use when they have to identify a musical excerpt in a pattern-matching task (e.g. (69; 70; 71).. However, this chunking process may be disrupted in the case of an unexpected or unconventional visual pattern in the notation, resulting in longer saccadic latency (3).

Moreover, music reading is not only a matter of pattern recognition (low-level processing), but also a matter of interpretation, which requires inferences from long-term memory ([LTM], i.e. high-level processing). Musicality and virtuosity might rely on a large musical knowledge structure (related to the musical situation model in LTM), combining all the information necessary for performing a musical score differently from using a computer, for example (15; 74). This extra-musical knowledge (outside the music score), representing the capacity of the experts, is composed of knowledge about the music style, the composer, the harmony and tonal rules, the fingering, or phrasing, etc. This might support the notion that musicians organize their practice and subsequent retrieval according to the formal structure of a piece of music (9; 10). These skills (chunking capacity and the building of a musical knowledge structure) have been acquired and memorized through extensive learning and practice (15; 48). Many theories of expert memory (11; 22; 31) explain how experts can mobilize a huge quantity of information very quickly, exceeding the limitations of working memory (WM). These theories usually propose that such individuals are capable of using a part of their LTM as WM. In this way, WM is conceived as a functional subset of LTM, which is represented at the brain level by specific neuronal connections (for a review, see (34) The development of an expert memory is supposed to build retrieval structures to facilitate the retrieval of information stored in LTM and use them strategically and efficiently to encode information in LTM based on cues that can later be reactivated to retrieve the information stored (24 ). All these theories are based on the three principles of Ericsson and Chase’s (21) skilled memory theory, which involves: 1) a specific encoding in a prior knowledge structure in LTM, such that information is encoded with elaborated cues related to a large body of relevant knowledge and patterns; 2) the use of retrieval structures to keep track of the information. These retrieval structures are separated LTM knowledge structures, hierarchically organized, in which the encoded information is associated with retrieval cues, activated at a later time to reinstate the conditions of encoding and retrieve the information; 3) an increase in encoding and retrieval performance, which speeds up with practice and training. The activity must be highly familiar. Experts can accurately anticipate demands for the retrieval of relevant information. Most expert memory theories have accorded a crucial role to both the organization of knowledge in memory and the retrieval structure with specific encoding in memory (21; 22; 23).

One of the theories that can be fitted to musician knowledge is that of long-term working memory ([LTWM]; (22). This model generalizes the retrieval structures in various domains of expertise to account for the large demands on working memory during text comprehension and expert performance. Ericsson and Kintsch (22) proposed two levels of encoding to attain reliable and rapid storage and access to information in LTWM, depending on the demand of the activity:

Our proposal for LTWM includes cue-based retrieval without additional encodings and cue-based retrieval with an elaborated structure associating items from a given trial or context. The demands a given activity makes on working memory dictate which encoding method individuals are likely to choose so as to attain reliable and rapid storage and access of information in LTWM. This encoding method, which is either a retrieval structure or an elaborated memory structure or a combination of the two, determines the structure of the acquired memory skill. (p. 220)

We suggest that LTWM may represent the ability of cross-modal integration for expert musicians.

### Music Reading and Cross-Modal Integration

Reading musical notation implies a transfer in visuo-spatial to tonal modality in WM (39). Music reading engages multimodality effects as musicians extract visual information from the musical score, interpret the musical structure and perform it in playing or singing, relying on their auditory feedback (52). The skill of musical audiation (i.e. hearing music while reading the score) is multimodal, integrative music reading observed in most highly expert musicians (7; 8). The auditory imagery of the future sound is activated while reading a musical excerpt (79). This ability to generate and use the auditory representation of the written music also underlies a simple visual pattern-matching task (28).. Training in musical visual notation shapes audio-visual integration while pairing symbols (53) and allows faster processing of tonal patterns (46). Thus, expertise in music reading seems to be related to a multisensory integration that evolves with training (55). Expertise in music reading also involves a sensorimotor transcription of the music’s spatial code (66). Reading and playing piano scores share and activate the same cortical areas (50).

Although previous research on eye movements and music reading has examined the characteristics of expertise, few studies have investigated cross-modal processing while reading and playing (1; 5; 19; 35; 58) and a capacity for cross-modal integration related to the musicians’ level of expertise (17, 18).

### Cross-Modal Reading and Playing Design

A cross-modal design was used to investigate visual processing in relation to the eye-hand span (58). Musicians were provided with an auditory model of a melody during a familiarization phase, before being asked to play this melody on the piano. The music material was a very simple, familiar children’s song. The “musically experienced adults” had to play the original or melodically altered version (one measure being shifted down a tone). This expectancy violation between the auditory representation and the corresponding notation caused a reduced eye-hand span in the melodically altered version. These findings could be due to the mismatch between the tune and written notation, or to the comprehension of the violation of tonal rules in an expert memory.

A cross-modal design was also used to investigate the comprehension of pitch relationships in written music (35). Pianists were asked to read and play either congruent or anomalous melodies at their own pace or with an external metronome. Anomalies in the notation led to processing difficulty, suggesting that proficient pianists integrate the music within the prior context. They show an incremental comprehension of pitch relationships during reading.

Although these experimental designs involved crossmodal processing difficulty for musicians, the findings were not discussed in terms of expert memory or crossmodal integration. Although previous research on eye movements and music reading has examined the characteristics of expertise, only a few studies have investigated the capacity for cross-modal integration related to musicians’ level of expertise.

### Amodal Integration for Skilled Musicians

In previous studies we attempted to extend the idea of amodality, acknowledged in relation to language and semantic memory (14; 37; 63; 65; 67) to the domain of music. There are two underlying hypotheses in cross-modal processing: (1) cross-modal conversion (the recoding hypothesis), whereby information in one modality is converted to the other modality; (2) cross-modal integration (the amodal hypothesis), whereby information is not encoded in a modality-dependent way, but is integrated at a higher level in an amodal representation. These two hypotheses operate at different information processing levels (perceptual for the former, conceptual for the latter) and depend on the individual’s prior knowledge and skill level in the activity or task to be carried out.

In an earlier experiment (5), we assumed that expert and non-expert pianists’ eye movements would highlight cross-modal integration only for expert musicians. In a piano sight-reading task, crossing visual and auditory information, most skilled musicians demonstrated a certain independence from the written score, making fewer fixations compared to nonexperts. They seemed to reactivate the auditory representation of the musical passage they had heard prior to reading. In contrast, less-skilled musicians were highly dependent on the written score and the input modality (making more fixations compared to experts). They could not benefit from the previous listening and had to build a new representation based on visual cues.

The relative independence of experts from the score was also verified when fingering written in the score was not adopted in execution. Fingering is an important visuo-motor cue that allows musicians to anticipate the position of their fingers on the instrument and find the optimal fingering combination for virtuosic playing (56). When expert pianists had previously heard the musical excerpt, they ignored difficult fingering annotated in the score and played using their own fingering. They applied a kind of memorized pattern, regardless of the input source. Conversely, non-expert pianists processed and applied the fingering written in the score, even if such fingering was not adapted for the performance (19).

Moreover, when expert and non-expert musicians had to decide whether a note was modified between the listened to versus the read versions of a musical fragment, the accent mark (a cue contributing to the phrasing prosody) appeared to constitute interference for non-experts. When the accent mark was incoherent, there were more incorrect judgments (18). These studies provide evidence that eye movements are not dependent only on the visual aspects of the score, but also on cognitive processes related to cross-modal integration in expert memory. Experts are able to encode musical information independently of the input modality and can retrieve it regardless of how the information is perceived (visually or auditorily). It follows that perceptual notation might be less important for experts, since they are capable of using their musical knowledge to compensate for missing (5; 64) or incorrect (19) information. Their knowledge of the musical structure, acquired with expertise, allows a relative independence from the written score for expert musicians. In cognitive terms, this means that an expert memory has developed over years of practice, allowing a huge range of musical knowledge storage. Moreover, this expert memory seems to facilitate the shift between vision and audition during music reading.

However, the issue of multisensory transfer across auditory and visual musical material processing has to be refined and more precisely tuned to determine whether cross-modal integration may be developed concurrently with an expert memory. Thus, the goal of this study was to investigate different levels of crossmodal information processing between non-expert (conversion) and expert (integration) musicians. From a cross-modal conversion perspective (recoding hypothesis), information in one modality is converted into the other modality, while from a cross-modal integration perspective (amodal hypothesis), a higher representation level (conceptual level) must be accessed to integrate information from various sources.

With the acquisition of musical expertise, multimodal processing (audio/vision) will evolve from a cross-modal conversion for non-experts to a cross-modal integration for experts. Our general hypothesis was thus that cross-modal integration for experts would be assigned to expert memory structures and to the inference processes afforded by prior knowledge and skill in music reading activity. Within this perspective, cross-modal integration between auditory and visual information for expert musicians will induce faster and more accurate processing (shorter first fixation duration [FFD] and dwell time [DT], a smaller number of fixations [NF] and fewer errors) than cross-modal conversion between auditory and visual information for non-experts (who will separate the two types of information in a cross-modal conversion – recoding hypothesis).

To investigate the different levels of cross-modal information processing and to be able to observe the cognitive processes engaged in music reading, ecological material introducing greater cognitive demands than in previous research was used (for details see the “Experimental Design” section and Table A1 in the Appendix). The classical musical excerpts were longer (8 measures) than in previous studies in order to overload the limitations of cognitive capacity for processing (51) in sequential presentation. All the music excerpts were written in the Western tonal tradition. They were always modified between the auditory (original version) and the visual version (modified version), with the modification either a violation of the tone mode or not (respecting or violating the musical tonal rules). Moreover, the excerpts were presented in sequential audio-visual presentation versus simultaneous audio-visual presentation. The purpose of the sequential presentation was to test the retrieval of information from memory, while the simultaneous presentation aimed to test cross-modal matching between the auditory and visual representation of notes. This task is not properly a sight-reading task (participants are not required to perform the music on the instrument), but rather a reading task, involving a match between sound and vision. This competence is one of the basic required competences in music learning and is trained in music conservatoires.

More precisely, the operational hypotheses attempting to test integration vs. conversion should be supported by the following interactions in terms of performance (errors) and time (FFD and DT):

1) Globally, experts should be more effective (performance and time) than non-experts, but this expertise should interact with the type of presentation (simultaneous vs. sequential). Simultaneous presentation, which requires the musician to match sound and vision very rapidly in order to detect the modified note, should be easier for experts than non-experts. As a consequence, we expect shorter FFD for experts compared to nonexperts. FFD entails earlier processing components, as it is based mostly on fast perceptual processes, such as recognition/identification (for reviews, see (for reviews, see (38 ; 59). Therefore, if experts use cross-modal integration, rather than conversion, they should be faster than non-experts in this simultaneous condition. FFD may reveal the immediacy of cross-modal integration processes and experts may integrate the two sources at first glimpse (FFD). Experts should also be faster with regard to DT, in which later processes are carried out regardless of the modification to the note.
2) We also expect an interaction between expertise and modification (violation vs. non-violation). Experts should be more effective (performance and time) than non-experts at detecting the modified note in the violation condition and thus present fewer errors and NF (performance), as well as faster times for DT and FFD (time). The same logic as above may be applied here. The matching between sound and note is faster in cross-modal integration and experts should be more effective than non-experts at detecting the modified note in the violation condition.
3) Concerning a three-way interaction between expertise, presentation and modification, experts should respond faster in the violation and simultaneous conditions than non-experts for FFD (immediate processing) as opposed to DT (late processing). In this more difficult condition (violation AND simultaneity), there should be no more processing for the following fixations and therefore no effects on DT. In contrast, non-experts who are unable to attain access to an amodal structure, should have longer DT for the modified note but no difference for FFD.

In brief, we hypothesize faster integration processes for experts when matching visual and auditory inputs simultaneously, modified by the complexity of the note violation.

Previous research has highlighted differences in cross-modal processing, contrasting expert and nonexpert musicians, but no studies have yet addressed how tonal rules may support cross-modal integration serving as retrieval cues in expert memory. To the best of our knowledge, no prior study has used eye-movement tracking to examine the effect of expert memory on the processing of simultaneous versus sequential presentation of cross-modal musical excerpts that respect (or not) the tonal rules of writing.

## Methods

### Participants

In total, 53 volunteer participants, comprising students, teachers and professional musicians, were recruited from the Conservatoire of Music in Nice. They gave their oral consent to participate in the experiment and they all had normal hearing and normal (or corrected-to-normal) vision. They were divided into two groups concerning expertise depending on their musical skills assessed according to their position in the musical institution. The sample thus comprised 26 experts (mean age 34 years, *SD*±13), with more than 12 years of academic practice in music, including teachers, professional musicians and final-year students, as well as 27 non-experts (mean age 15 years, *SD*±3), with 5–8 years of academic practice in music, comprising students at the Conservatoire of Music in Nice, France. All the participants were assigned to all the experimental conditions. The required sample size for *F*-tests (repeated measures analysis of variance [ANOVA], within-subjects factors) was estimated using a power analysis (GPower 3.1.7) (25). Using a full within-factors design (see below) and 20 trials, the power analysis predicted 22 participants to be sufficient to reach a significance level of *p*=.01 (power=.99; effect size=.25). Thus, the current sample was well above the sample size required.

### Experimental Design

**Figure 1:**
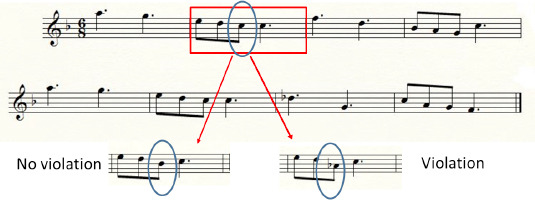
Example of the two types of note modification for a musical excerpt: No violation (same tone mode) versus Violation (violation tone mode).

To implement this study, we needed to use non-artificial material that was not too simple. The material was chosen to be representative of the music used by musicians in ecological conditions of musical practice. Taking into account that there are more fixations with more note symbols in the score and fixation durations and DTs are shorter at a faster tempo, both (number of notes and tempo) were varied across the melodies. Thus, 20 excerpts 8 measures in length were selected from the tonal repertoire of classical music. Each melody consisted of a single music stave written in the treble clef. The 20 melodies of various levels of difficulty were composed of 18 to 58 notes (*M*=37, *SD*±11.60), written in various time signatures (2/4, 3/4, 4/4, 6/8) and presented in a tempo from 60 to 120 bpm. The characteristics of the 20 melodies, namely tempo, time signature and number of notes, are given in Table A1 in the Appendix). The violation factor introduced in reading entailed constructing two versions of each excerpt, one with a tone violation (violation of the tonal mode, V) and one with no such violation (modified note staying within the same tonal mode, NV). For the tone violation condition, one note was modified by a semitone or a tone in each musical excerpt and thus a trailing sharp or a flat symbol was added to the written notation in the score, unlike in the no violation condition (an example is presented in Figure 1). The modification of the note could occur in any one of the 8 measures. Each stave was created using the Finale software™. For the auditory presentation, the 20 original excerpts were converted to .wav format and their duration varied across the excerpts in the range 11–23 s according to the number of notes. Sequential (decoupled listening and reading) versus simultaneous (coupled listening and reading) cross-modal presentations of these randomized excerpts were displayed on a computer.

The experiment, displaying the audio-visual version of the musical stimuli, was designed using SMI Experiment Center™. A Latin square design was applied to counterbalance each melody across conditions and participants (which gave four lists of stimuli). Each participant was assigned to one of the four lists, such that each melody was presented once to each participant and the four conditions in which melodies were presented (e.g., violation or not, simultaneous or sequential) were manipulated between participants. For each participant, the list of 20 melodies consisted of 5 examples for each of the 4 combinations of conditions. Serial order of condition was randomized within each participant.

### Apparatus and Calibration

During recording, participants were comfortably seated in a quiet room at the Conservatoire. After a nine-point phase of calibration, accepted if the average error was less than 0.5° of the visual angle, their eye movements were sampled at a frequency of 500 Hz using the SMI RED 500™ eye-tracking system. The music stave stimuli were presented on a 17” monitor with an image resolution of 1280 x 1024 pixels at a viewing distance of 60 cm. Sony Plantronics™ headphones were connected to the computer to display auditory excerpts as stimuli for the purpose of simultaneous and sequential presentation.

### Procedure

**Figure 2:**
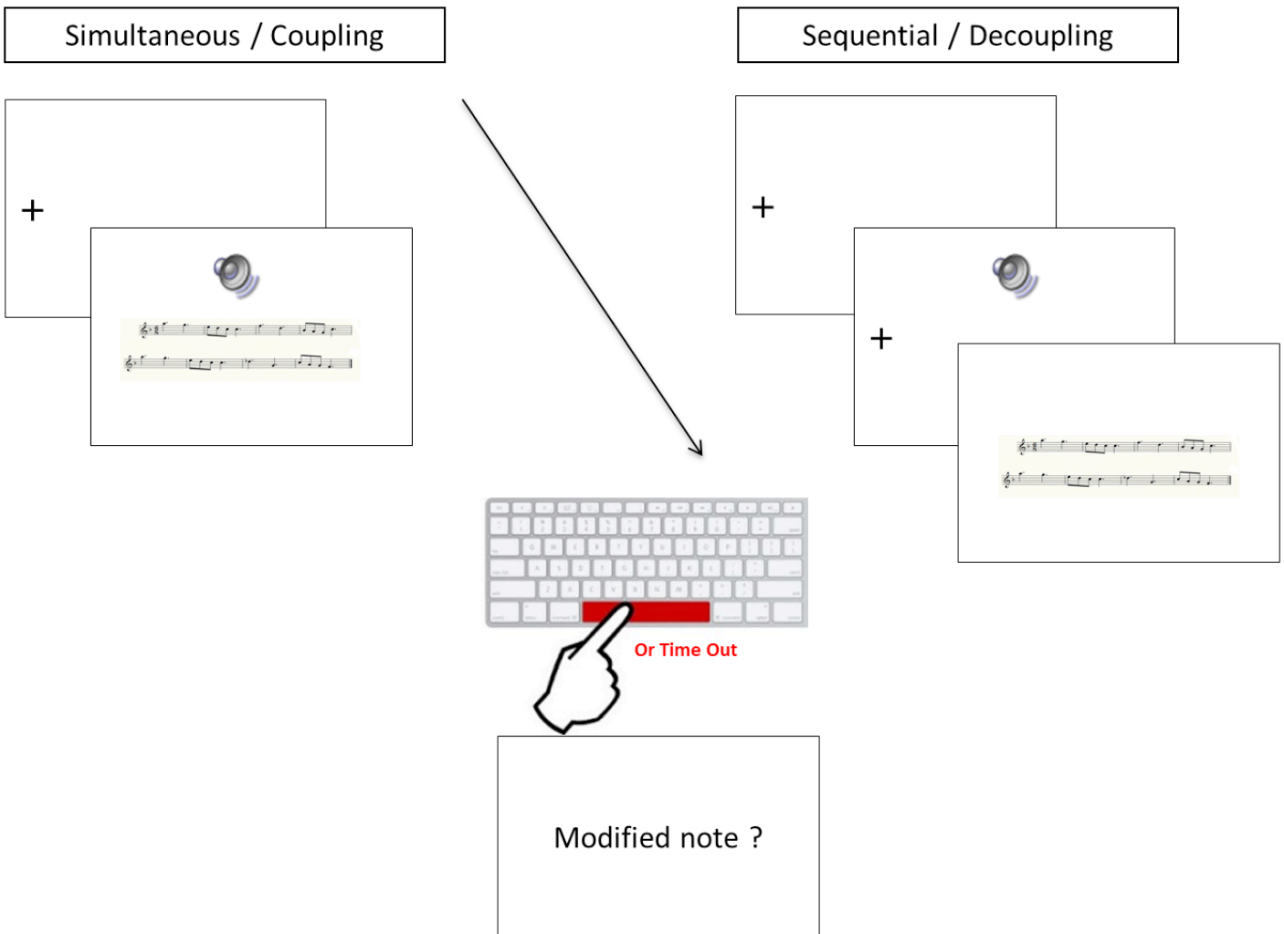
Design of the experimental procedure for the two conditions of auditory and visual excerpt presentation: (simultaneous/coupled and sequential/decoupled).

The participants were instructed to 1) detect as fast as possible a modified note between the heard (tune) and read (written) excerpts of music and 2) report aloud the name of the detected modified note. The participants fixated on a cross within a trigger area of interest (AOI). As soon as their eyes were detected on the trigger AOI, the musical excerpt was presented in two randomized cross-modal conditions: listening to the fragment prior to reading (sequential, decoupled) or listening to the fragment while reading (simultaneous, coupled). As soon as they found the modified note, the participants pressed the space bar of the keyboard, triggering the presentation of a new screen that asked them to report the name of the modified note that was displayed. The time out was fixed from 11 s to 23 s according to the duration of the excerpt presentation (see Table A2 in the Appendix). The experimenter wrote the name of the detected note on a digital tablet, then pressed the space bar of the keyboard to display the next trial. The session lasted 30–40 min for each participant. Example trials are illustrated *Figure 2*, showing the design of the experimental procedure.

### Data Analyses

Data from the 53 participants were included in the detection error and eye-tracking analyses.

The detection error rate was calculated by finding the ratio between the number of errors and the number of items (20 excerpts). The detection error rate was submitted to a 2 x 2 x 2 repeated-measures ANOVA with 1 between-subjects factor: musical expertise (experts / non-experts) and 2 within-subjects factors (2 levels each): auditory/visual presentations (sequential-decoupling vs. simultaneous-coupling) and modification (violation tone mode vs. no violation tone mode).

**Figure 3:**
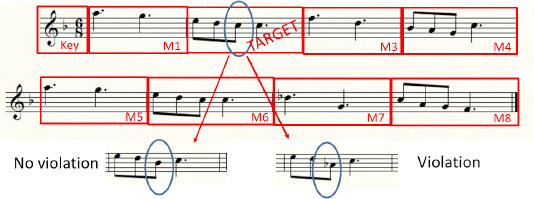
Example of AOIs designed for global analyses for a musical excerpt: key signature, Measure 1 (M1), Target measure (TARGET) with violation tone mode or no violation tone mode and Measure 3 to 8 (M3-M8). Thus, in this example the label of measure 2 is replaced by “TARGET” because that is the measure in which the modification occurred.

For eye-movement analyses, each musical excerpt, composed of 8 measures, was divided into 9 AOIs, respectively corresponding to the target measure (in which the modification of the note occurred), the key signature area and the remaining seven measures of the musical excerpt (as presented in the example in *Figure 3*). Considering that, over all 20 excerpts, the modification of the note occurs in one of the 8 measures and considering that the label of the measure in which the note is modified is replaced by “Target”, a total of 10 AOIs results (the key signature area, the target AOI and the 8 measures, over all 20 excerpts). Eye movements were detected from the raw eye coordinate data in SMI Begaze software™ using a velocity-based algorithm. The eye-movement analyses included the FFD, NF and DT. Fixations with a duration less than 100 ms were excluded. Moreover, analyses of eye-movement metrics were carried out at a global and a local level.

At a global level (i.e. testing the effects of modification over the whole score and over the 20 excerpts), analyses were done for every AOI and the eye-movement metrics were submitted to a 2 x 2 x 2 x 10 repeated measures ANOVA with 1 between-subjects factor: musical expertise (experts/non-experts) and 3 within-subjects factors: auditory/visual presentations (sequential/decoupled vs. simultaneous/coupled), modification (violation tone mode vs. no violation tone mode) and 10 AOIs (the target, key signature and 1 to 8 measures over all 20 excerpts).

**Figure 4:**
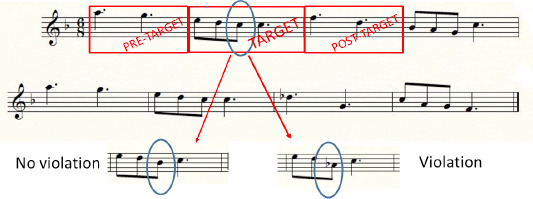
Example of AOIs designed for local analyses for a musical excerpt: PRE-TARGET, TARGET (with violation tone mode or no violation tone mode) and POST-TARGET measures.

**Figure 5:**
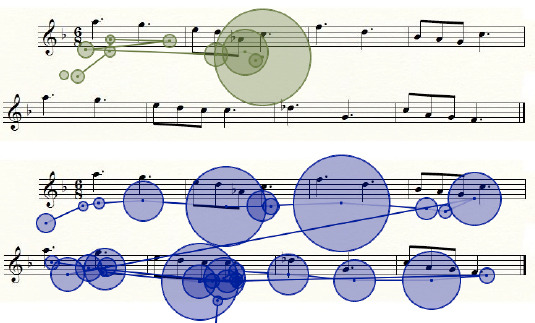
Example of visualization for one expert’s eye tracking scanpath (above) and one non-expert’s eye-tracking scanpaths (below) on the same musical excerpt and experimental conditions (simultaneous presentation and tone violation). Each color represents a different participant.

At a local level (i.e. attempting to investigate the effects of modification on the AOI target and focusing only on pre-target, target and post target AOIs), analyses were carried out to contrast experimental conditions only into three AOIs: 1) the target measure in which the note was modified, 2) the pre-target and 3) the post-target measures, as presented in the example in *Figure 4*. In this example, as the target is localized in the second measure, the pre-target is localized in the first measure and the post-target is localized in the third measure, but of course, pre and post-target differed as function of the target position on the stave. Thus eye-movement metrics were submitted to a 2 x 2 x 2 x 3 repeated measures ANOVA with 1 between-subjects factor: musical expertise (experts/non-experts) and 3 within-subjects factors: auditory/visual presentations (sequential/decoupled vs. simultaneous/coupled), note modification (violation tone mode vs. no violation tone mode) and 3 AOIs (pre-target, target and post-target). The *p*-values were corrected according to the Greenhouse–Geisser procedure and the post-hoc comparisons were done with the Newman–Keuls test. Example of visualizations for experts’ and non-experts’ eye-tracking scanpaths are shown in *Figure 5*.

## Results

Overall average values for the detection error rate and eye movements (First Fixation Duration – FFD and Dwell Time - DT), both whole score and the target measure, are summarized in Table A and Notes:. Global analysis reveals a time course analysis over time, while local analysis focuses the processing on only the critical parts of the score.

**Table A:** Results for detection error rate and eye movements (Gobal and Local analyses).

**Detection Error** **Rate**		Sequential		Simultaneous	

		NV	V	NV	V
Errors (%)	NE	63.70 (4.52)	55.93 (5.28)	27.03 (4.83)	36.67 (4.23)
	E	45.90 (4.61)	21.92 (5.38)	8.11 (4.92)	9.10 (4.31)

**Eye movements** **Global**		Sequential		Simultaneous	

		NV	V	NV	V

FFD (ms)	NE	286 (37)	310 (36)	302 (34)	311 (40)
	E	264 (37)	264 (37)	260 (35)	284 (41)

DT (ms)	NE	1313 (195)	1389 (171)	1204 (148)	1331 (155)
	E	1217 (198)	1199 (174)	938 (151)	1052 (158)

**Eye movements** **Local**		Sequential		Simultaneous	

		NV	V	NV	V

FFD (ms)	NE	298 (22)	331 (29)	393 (30)	370 (40)
	E	248 (22)	293 (30)	267 (30)	308 (41)

DT (ms)	NE	1906 (112)	2626 (186)	3057 (192)	3623 (278)
	E	2282 (115)	2777 (190)	2010 (196)	2348 (283)

**Table B:** Statistics for DT on the target and post-target (errors as co-variate)

		Target	Post-Target
Sequential	NV	ns	ns
	V	ns	ns

Simultaneous	NV	*F*(2, 50)=7.86 *p*=.001	*F*(2, 50)=10.18 *p*<.001
	V	*F*(2,50)=4.45 *p*=.01	*F*(2, 50)=14.68 *p*<.001

Table A2 (in the Appendix) summarizes the results of ANOVAs for the detection error rate, FFD (the duration of the first fixation that hit an AOI), DT (the sum of durations from all fixations and saccades that hit an AOI) and NF (the number of fixations within an AOI). As DT and NF were positively correlated, *r*(51)=.79, *p*<.05, thus reflecting a similar construct, we omit NF from this section (although they are included in Table A2 in the Appendix).

### Detection Error Rate

The analysis of the detection error rate indicated main effects of expertise (*F*(1, 51)=35.19, *p*<.001, η^2^=.41), presentation (*F(*1, 51)=59.89, *p*<.001, η^2^=.54) and modification (marginally significant; *F*(1, 51)=3.51, *p*=.067, η^2^=.06), with a lower error rate for experts (21%) compared to non-experts (46%), in simultaneous presentation (21%) compared to sequential presentation (47%) and in violation (31%) compared to no violation (36%). The interaction between expertise and modification was significant (*F*(1, 51)=4.85, *p*=.032, η^2^=.09), showing a lower error rate for violation (16%) compared to no violation (27%) only for experts (*F*(1, 51)=8.15, *p*=.006). The comparison between violation (46%) and no violation (45%) was not significant for non-experts (see Table A and Notes:).

The correlation between the duration of audio recordings and number of errors was calculated to verify that the error rate was not dependent on audio duration (11 to 23 s). There was a weak positive correlation in the sequential condition (*r*(530)=.09, *p*=.028). However, there was no correlation in the simultaneous presentation (*p*=.205, ns) or according to expertise (experts: *p*=.103, ns; non-experts: *p*=.140, ns). This trivial effect is related to the number of notes on the score. When there are more notes, memory may be overloaded (that was the goal of the study), leading to more errors.

**Figure 6:**
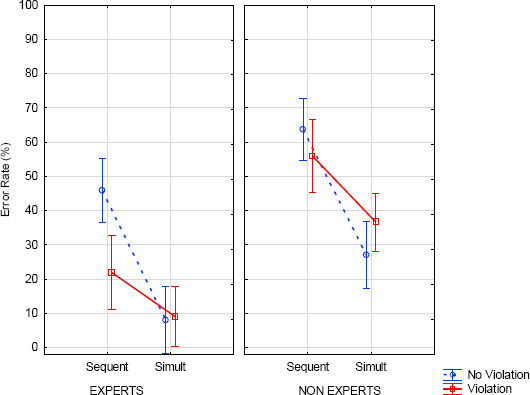
Error rate (%) according to expertise, note modification and presentation (error bars represent standard error).

The interaction between presentation and modification (*F*(1, 51)=13, *p*<.001, η^2^=.20) showed a higher detection error rate with sequential presentation in the no violation condition (55%) compared to the violation condition (39%), with *F*(1, 51)=18.30, *p*<.001, while in simultaneous presentation this comparison was not significant (no violation 18%; violation 23%). The three-way interaction between expertise x presentation x modification was not significant (see *Figure 6*).

### Eye Movements: Global Analysis

**Figure 7:**
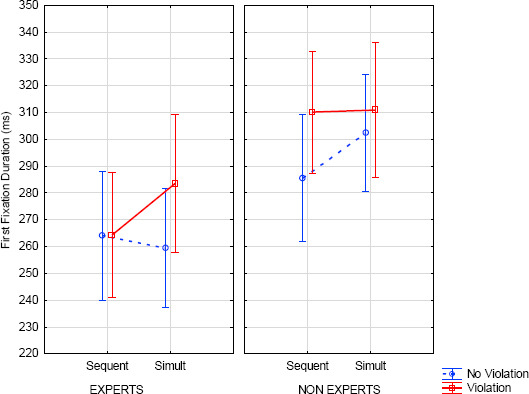
First fixation duration (ms) according to expertise, note modification and presentation (error bars represent standard error).

The analysis of FFD indicated main effects of expertise (*F*(1, 51)=6.71, *p*=013, η^2^=.12) and modification (*F*(1, 51)=8.33, *p*=.006, η^2^=.14) with shorter FFD for experts compared to non-experts and lower FFD in the no violation condition compared to the violation condition. Both were qualified by a significant interaction between expertise x modification x presentation (*F*(1, 51)=3.95, *p*=.052, η^2^=.07). This three-way interaction (see *Figure 7*) showed longer FFD in the sequential presentation with violation (compared to no violation) for non-experts (*F*(1, 51)=11.46, *p*=.01). Conversely, FFD was longer in simultaneous presentation, with violation (compared to no violation) and for experts (*F*(1, 51)=3.89 *p*=.054). In addition, there was a main effect of AOIs (*F*(9, 459)=22.78, *p*=.001, η^2^=.31), showing more fixations on the target measure compared to others (*F*(1, 51)=33.39, *p*<.001). There was also a significant interaction between AOIs x presentation (*F*(9, 459)=2.71, *p*=.01, η^2^=.05). No other effects were significant (see Notes: and Table A, *Table A2* in the Appendix).

**Figure 8:**
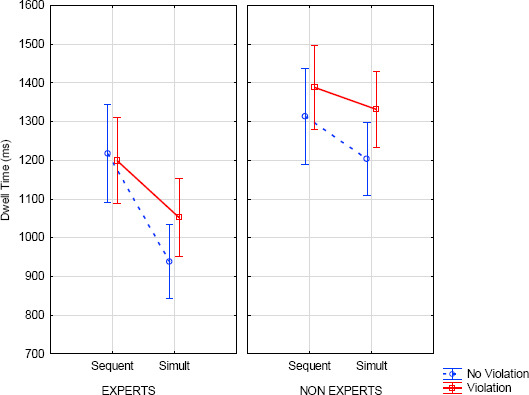
Dwell time (ms) according to expertise, note modification and presentation (error bars represent standard error).

The analysis of DT (ms) indicated main effects of expertise (E < NE; *F*(1, 51)=11.98, *p<*.001, η^2^=.19) and presentation (sequential > simultaneous; *F*(1, 51)=27.88, *p*<.001, η^2^=.35). Both were qualified by an interaction between expertise and presentation (*F*(1, 51)=5.34, *p*=.025, η^2^=.10), showing shorter DT in simultaneous presentation compared to sequential presentation for both experts (*F*(1, 51)=28.28, *p*<.001) and non-experts (*F*(1, 51)=4.49, *p*=.039). Moreover, for simultaneous conditions, DTs were shorter for experts compared to non-experts (*F*(1, 51)=21.11, *p*<.001). There was also a main effect of the modification (violation > no violation; *F*(1, 51)=9.40, *p*=.003, η^2^=.16). A main effect of AOIs (*F*(9, 459)=121.95, *p*<.001, η^2^=.70) showed longer DT on the target measure compared to others (*F*(1, 51)= 242.26, *p<*.001). AOIs interacted with expertise (*F*(9, 459)=2.70, *p*=.005, η^2^=.05), with presentation (*F*(9, 459)=6.92, *p*<.001, η^2^=.12) and with modification (*F*(9, 459)=7.17, *p<*.001, η^2^=.12) (see Table A2 in the Appendix). The three-way interaction between AOIs x presentation x expertise (*F*(9, 459)=7.03, *p*<.001, η^2^=.12) showed shorter DT in sequential presentation (compared to simultaneous) on the target measure (compared to other AOIs) for non-experts only (*F*(1, 51)=21.03, *p*<.001). This comparison was not significant for experts (*F*(1, 51)=1.93, ns) (see Notes: and Table A, *Table A2* in the Appendix). The three-way interaction between expertise x presentation x modification was not significant (*F*(1, 51)=.51, ns) (see *Figure 8*).

Moreover, to resolve a possible confounding effect between age and expertise, a multivariate analysis of covariance (MANCOVA) was carried out on DT, which showed that there was no effect of age (co-variate) on any experimental condition (all *p*>.05). This means that the main effect and interactions that we have presented are independent of age and only dependent on expertise.

### Eye Movements: Local Analysis

The local analysis focused on the pre-target, target (where the note modification occurred) and post-target AOIs (see Notes: and Table A, *Table A2* in the Appendix).

The FFD analysis indicated main effects of expertise (E < NE; *F*(1, 46)=6.34, *p=*.015, η^2^=.12) and AOIs (*F*(2, 92)=15.16, *p<*.001, η^2^=.25). There was longer fixation for the target than either the post-target (*F*(1, 46)=11.56, *p=*.001) or the pre-target AOIs (*F*(1, 46)=6.55, *p=*.014). There was longer fixation for the post-target than the pre-target AOIs (*F*(1, 46)=24.29, *p<*.001). No other effects were significant.

**Figure 9:**
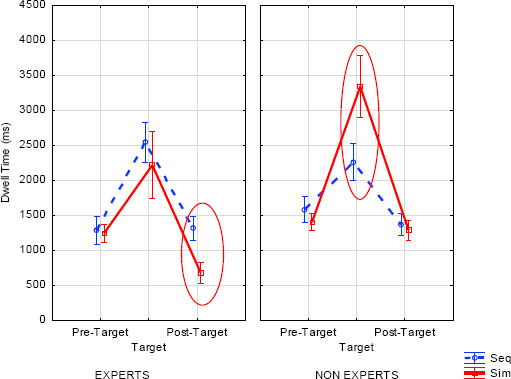
Dwell time (ms) for the pre-target, target and post-target AOIs according to expertise and presentation (error bars represent standard errors).

The DT analysis showed main effects of expertise (E < NE; *F*(1, 46)=10.78, *p*=.002, η^2^=.19), modification (no violation < violation; *F*(1, 46)=6.40, *p*=.015, η^2^=.12) and AOIs (*F*(2, 92)=185.40, *p*<.001, η^2^=.80). There was longer fixation for the target than either the pre-target (*F*(1, 46)=193.34, *p*<.001) or the post-target AOIs (*F*(1, 46)=221.70, *p*<.001). There was longer fixation for the post-target than the pre-target AOIs (*F*(1, 46)=19.23, *p*<.001). Modification interacted with AOIs (*F*(2, 92)=25.78, *p*<.001, η^2^=.36), showing longer DT on the target (*F*(1, 46)=26.51, *p<*.001) and shorter DT on the post-target for the violation tone mode (*F*(1, 46)=10.63, *p*=.002), compared to no violation. The comparison was not significant for the pre-target (*F*(1, 46)=.78, ns). Presentation interacted with expertise (*F*(1, 46)=22.19, *p*<.001, η^2^=.33), showing that simultaneous presentation induced longer DT for non-experts (*F*(1, 46)=9.59, *p=*.003) but shorter DT for experts (*F(*1, 46)=12.61, *p*=.001), compared to sequential presentation. Presentation interacted with AOIs (*F*(2, 92)=9.80, *p*<.001, η^2^=.18) showing, for simultaneous presentation, longer DT on the target (*F*(1, 46)=4.78, *p*=.034) but shorter DT on the post-target (*F*(1, 46)=20.30, *p<*.001), compared to sequential presentation. The difference was not significant for the pre-target (*F*(1, 46)=2.56, ns). The three-way interaction (Figure 9) between AOIs x presentation x expertise (*F*(2, 92)=10.52, *p*<.001, η^2^=.19) showed that for experts, DT was shorter in simultaneous presentation (compared to sequential) on the post-target only (*F*(1, 46)=29.33, *p*<.001); the comparisons were not significant for the pre-target (*F*(1, 46)=.17, ns) or the target (*F*(1, 46)=1.68, ns). For non-experts, DT was longer in simultaneous presentation (compared to sequential) on the target only (*F*(1, 46)=21.53, *p<*.001); the comparisons were not significant for the pre-target (*F*(1, 46)=3.67, ns) or post-target (*F*(1, 46)=.59, ns).

Furthermore, we tried to see whether DT on the AOIs might be dependent on success in detecting the modified note (i.e. number of errors). As we note above, experts made significantly fewer errors than non-experts, but the question is still open as to whether they remembered the note as soon as they detected the modification. For this purpose, we carried out a MANCOVA on DT with errors as the co-variate in any experimental condition. DT was significantly shorter on the target and post-target AOIs in the simultaneous conditions for experts compared to non-experts. There were naturally no effects on the pre-target.

This result complements the previous data showing that experts are able to detect the modification immediately in the simultaneous condition and adjust their eye movements accordingly on the post-target (fewer fixations).

## Discussion

The aim of our study was to investigate the cross-modal integration hypothesis for expert musicians related to the development of expert memory. It was hypothesized that with the acquisition of musical expertise, multimodal processing (audio/vision) would be faster for experts due to cross-modal integration than for non-experts, who it was supposed would use a cross-modal conversion. The study compared the performance of expert and non-expert musicians in detecting a modified note in cross-modal presentation. The music excerpts were presented in a sequential audio-visual presentation versus simultaneous audio-visual presentation. The versions of music were modified between the auditory (original version) and the visual version (modified version), respecting or violating musical tonal rules.

Several main effects of expertise, presentation and note modification were found in the global and local analyses. For most of the metrics (errors, FFD, DT), the results show that experts performed faster (shorter FFD and DT) and more accurately (fewer errors) than non-experts. These results are consistent with previous research on expertise and music reading (17, 18; 61).

Moreover, the sequential condition proved to be more difficult (longer DT and more errors) than the simultaneous condition. This difference in DT may be explained by the constraints of 1) working memory (the sequential presentation entails remembering the music before detecting the modification visually) and 2) the sound flow during simultaneous presentation. Reading tempo is, of course, driven by the sound because the eyes want to match what they hear (45). In addition, there was longer fixation on the target AOI (FFD, DT) than the other AOIs and longer fixation than for the pre- and post-target, both at the perceptual (FFD) and processing (DT) levels. This suggests an immediate detection of the modified note. The modified note is detected faster when there is no tone violation, compared to a tone violation (shorter FFD and DT), but this produces more errors (suggesting a speed-accuracy trade-off strategy). As Janata and Reisberg (42 ) pointed out, this effect on DT may be determined by the relative position within the tonal hierarchy. Moreover, it has been shown that syntactic incongruities in both musical and linguistic material increase the proportion and duration of fixations (1).

More concretely, to support our hypothesis of cross-modal integration for experts, we expected three types of interaction (see Introduction). First, we expected an interaction between expertise and the type of presentation (simultaneous vs. sequential) in the influence on DT. This was the case: experts processed both the simultaneous and sequential encoding faster than non-experts (shorter DT). Moreover, experts detected the modified note faster, especially in the simultaneous condition, in which cross-modal integration may be applied. This effect is particularly striking on the target AOI, for which the modification appeared, and the post-target AOI. Experts presented faster DT on the post-target in the simultaneous condition than in the sequential presentation (while there was no such difference either for the pre-target or target). One explanation may be related to the immediacy of processing; in the simultaneous condition, once they had found the modification in the target measure, they went further, following the sound flow, involving less processing. In contrast, in the sequential condition, such sound flow was not present during reading (because it was given previously) and the time delay might reflect the time required to access that memorized sound. However, we did not observe this interaction for FFD. A possible explanation is that, of course, the modification is not taken into account in this interaction and the absence of an effect on FFD may be due to the lack of any difference between experts and non-experts on the modification factor. In this case, violation and non-violation are collapsed together and we expected a stronger effect in the violation condition. This rationale is supported by the three-way interaction described below.

Second, we expected an interaction between expertise and modification. This was the case for errors: the violation was detected better by experts than non-experts. This means that experts processed better the type of notes that were modified, especially in the violation condition. They were better at detecting the modified note in the violation condition (16%) compared to no violation (27%), while there was no difference for non-experts (46% vs. 45%). This is probably because of the construction of an expert memory over the experts’ many years of practice. For experts, tonal rules probably supported cross-modal integration, serving as retrieval cues in expert memory (17, 18). However, this advantage concerning performance was not associated with faster fixations (no effect of FFD and DT).

Finally, we had a three-way interaction between expertise, presentation and modification in the influence on FFD but not on DT. As expected, experts were more effective in the violation and simultaneous conditions than non-experts on FFD (immediacy of processing – earlier processes) rather than on DT (late processes). This effect reveals the immediacy of the cross-modal integration processes for experts and shows that they are able to integrate the two sources on the first look (FFD). This effect supports our cross-modal integration hypothesis for experts, assumed to be faster than a cross-modal conversion process as it avoids the tedious conversion between sound and vision. Moreover, there was no effect on DT for experts, meaning no additional processing occurred in the late fixations on the target and post-target. In contrast, non-experts, who were likely unable to gain access to an amodal structure, showed longer DT on the modified note, but no difference on FFD. Thus, in the simultaneous condition, experts detected the violation immediately when it was encountered, while non-experts ignored it. Once again, this might be the consequence of a better structure of music knowledge that allows quick access to the tonal rules.

Taken together, all these findings show that experts are more capable of detecting the modification than non-experts, either with faster processing (FFD, DT) or better performance (errors). Each dependent variable provides evidence of the superiority of experts in the task. These results support the hypothesis that the main difference between experts and non-experts was due to the difference in knowledge structures in memory (as postulated by LTWM theory; (4), built over time with practice. It also suggests that these high-level knowledge structures in memory contain harmonic and tonal rules. Another argument consistent with this idea is the fact that simultaneous presentation appears to allow faster processing for experts than sequential presentation (DT). According to our cross-modal theory, it would seem that these knowledge structures related to music are amodal for experts, avoiding the need to convert the auditory information into visual information in detecting the violation. In contrast, the non-availability of such amodal structures for non-experts may explain their longer processing time, suggesting a kind of recoding process to carry out the cross-modal matching to detect the violation. Following LTWM theory (22), music knowledge might be stored as a retrieval structure, allowing faster access to the information stored in memory from musical cues (73; 75).

Our data clearly extend our past results (16, 17, 18; 19). This model suggests that experts organize their knowledge into so-called “retrieval structures” in long-term memory (LTM) in order to develop efficient retrieval strategies that surpass short-term memory (STM) capacities (12; 22; 29).. Based on this model, one can assume that musical knowledge structures are directly activated by visual, auditory and motor retrieval cues (73). The LTWM model seems appropriate for explaining how expert musicians efficiently manage the constraints of music reading and execution. In music cognition studies, inter-individual differences between expert and non-expert musicians have been discussed in terms of specific encoding and retrieval strategies in memory (15; 5; 75). These strategies allow the efficient retrieval and recycling of knowledge stored in memory using retrieval cues for efficient processing of the musical information and practice (2; 5; 75). In the classical music tradition, tonal musical writing is driven by tonal and harmonic rules and codifications (13), which seem to belong to the retrieval mechanisms described above. Empirical studies have observed classical concert pianists preparing their musical performance, using the musical structure to organize the performance and memorize a new piece of music in only a few hours of training (9; 40). The results of the present study argue in favour of different processing of cross-modal information with an increase in musical expertise. This ability seems to evolve from a cross-modal conversion for non-experts to a cross-modal integration for experts, with integration at a higher level, in an amodal representation. This ability might be related to the construction of an expert memory, confirming amodal integration for expert musicians (18).

However, one can argue that the process cannot be modelled finely based only on time variables. Different interpretations may be possible: 1) a cross-modal integration process as hypothesized, or 2) a very fast and effective conversion process, from visual to auditory or vice versa, or 3) some combination of both of the above. Our goal here was to bring elements and outline data consistent with this cross-modal integration hypothesis for expert musicians. In addition, some questions remain open: sensory inputs should be segregated when they come from different sources, so how then do musicians’ brains integrate sensory inputs into a coherent and unified perception? How does multisensory integration emerge during the development of expertise in music reading?

One possibility to disentangle between integration and conversion processes would be to get more fine-grained metrics of time or activation levels combining, for example, eye tracking with brain techniques (ERPs, fNIRS, fMRI) that measure cortical areas. To go further and go beyond the limits of the eye-tracking method, these questions may be investigated using eye movement-related potentials ([EFRPs]; (5), which may allow us to disentangle the different processes occurring during fixation. For example, by comparing novice and expert music readers, (76)) showed a category selectivity for musical notation (compared to Roman and pseudo letters) observed in the first ERP component (C1), evoked 40–60 ms after stimulus onset, for experts.

The question concerning the role of expertise in the processing and integration of multisensory information is very interesting and indeed is a crucial question for music teaching, both in terms of training young musicians to listen to what they are reading on the score and also emphasizing the need for deliberate practice, which participates in the construction of musical expertise. The field of research requiring further investigation of eye tracking and music reading is huge and innovative methods should be developed. For example, using a machine-learning technique (advanced multivariate pattern analysis [MVPA]), we recently classified musicians based on their visual performance while reading a musical score (fixation duration, saccade amplitude, pupil dilation) (4). MVPA has been used successfully in cognitive neuroscience to infer the content of representations encoded in patterns of cortical activity from functional neuroimaging data (O’Toole et al., 2007). It has also been successfully applied to eye-movement data to classify the viewer and the visual stimulus (33) or the task (36). Applying MVPA in music reading uses the same logic to investigate whether the eye-movement record contains sufficient information to permit inferences about the music reading that a person is engaged in – and by extension to the person’s underlying expertise.

## Ethics and Conflict of Interest

We declare that the contents of the article are in agreement with the ethics described in http://biblio.unibe.ch/portale/elibrary/BOP/jemr/ethics.html and that there is no conflict of interest regarding the publication of this paper.

## Appendix

**Table A1:** Characteristics of the 20 applied melodies: tempo, time signature, number of notes and duration of visual presentation corresponding to the duration of auditory presentation (in sequential and simultaneous presentation).

Score	Tempo	Time Signature	Number of Notes	Sequential and Simultaneous Presentation Duration (ms)
1	120	2/4	32	13000
2	120	2/4	25	21000
3	100	2/4	27	15000
4	100	2/4	36	15000
5	100	6/8	48	20000
6	100	2/4	15	23000
7	120	6/8	24	18000
8	100	3/4	36	20000
9	60	2/4	58	21000
10	90	3/4	44	21000
11	120	4/4	28	21000
12	60	2/4	41	22000
13	120	4/4	43	22000
14	120	3/8	18	11000
15	80	2/4	50	17000
16	60	2/4	46	21000
17	80	2/4	29	17000
18	120	3/4	25	17000
19	90	2/4	44	16000
20	70	2/4	39	19000

**Table A2:** ANOVA results.

DV	Effect	F	p	η^2^	Planned Comparisons
**Detection Error Rate**	EXPERTISE	35.19	.001	.41	E<NE
	PRESENTATION	59.89	.001	.54	Sim < Seq
	MODIFICATION	3.51	.0 7	.0	Viol < No Viol
	EXPERTISE X MODIFICATION	4.87	.032	.09	If E : viol < No Viol (.01) ; If NE viol = No Viol (ns)
	PRESENTATION X MODIFICATION	13	.001	.20	If Seq : No Viol > Viol (.001); If Sim : No Viol = Viol (ns)
**First Fixation Duration (ms)**	GLOBAL ANALYSIS				
	EXPERTISE	.71	.013	.12	E<NE
	MODIFICATION	8.33	.006	.14	Viol > No Viol
	EXPERTISE X MODIFICATION X PRESENTATION	3.95	.052	.07	If NE & if Seq : Viol> No Vio; If E & If Sim : Viol > No Viol
	AOIs	22.78	.001	.31	Target > others (.001)
	AOIs X PRESENTATION	2.71	.004	.05	Seq > Sim on M7; ns on Target, Key, M1, M2, M3, M4, M5, M 6, M8
	LOCAL ANALYSIS				
	EXPERTISE	.34	.015	.12	E<NE
	AOIs	15.1	.001	.25	Target > Post-Target (.001); Target > Pre-Target (.014); Post-target > Pre-Target (.001)
**Dwell Time (ms)**	GLOBAL/ANALYSIS				
	EXPERTISE	11.98	.001	.19	E<NE
	PRESENTATION	27.88	.001	.35	Sim < Seq
	MODIFICATION	9.40	.003	.1	Viol > No Viol
	EXPERTISE X PRESENTATION	5.34	.025	.10	If Sim, E < NE (.001); If Seq, E < NE (.054); Sim < Seq for E (.001) & NE (.039)
	AOIs	121.95	.001	.70	Target > others (.001)
	AOIs X EXPERTISE	2.70	.005	.05	E<NE on Target (.021), M1 (.006), M2 (.013), M5 (.01 6), M7 (.001); Key, M3, M4, M5, M8 (ns)
	AOIs X PRESENTATION	.92	.001	.12	If Target, Seq<Sim (.031) ; if others AOIs Seq > Sim (.001)
	AOIs X PRESENTATION X EXPERTISE	7.03	.001	.12	If NE : Seq < Sim on Target vs others AOIs ; if E (ns)
	AOIs X MODIFICATION	7.17	.001	.12	If Target, Viol > No Viol (.001), if M4 Viol < No Viol (.001), if Key, M1, M2, M3, M5, M 6, M7, M8 (ns)
	LOCAL/ANALYSIS				
	EXPERTISE	10.78	.001	.18	E<NE
	EXPERTISE X PRESENTATION	22.18	.001	.33	if E : Seq > Sim (.001); If NE : Sim > Seq (.003)
	MODIFICATION	.40	.015	.12	Viol > No Viol
	AOIs	185.40	.001	.80	Pre-Target > Post Target (.001); Target > pre-Target (.001); Target > post-Target (.001)
	AOIs X PRESENTATION	9.80	.001	.18	If pre-Target Seq = Sim (ns); if Target seq < Sim (.034); if Post-Target Seq > Sim (.001) If E, if pre-Target or Target seq = sim (ns); if post-Target Seq > sim
	AOIs X PRESENTATION X EXPERTISE	10.52	.001	.19	(.001) If NE if pre-Target seq = >sim (.062); if Target Seq < sim (.001); if post-Target Seq = sim (ns)
	AOIs X MODIFICATION	25.78	.001	.3	If Pre-Target No Viol = Viol (ns); if Target Viol > No Viol (.001), If PostTarget No Viol > Viol (.002)
**Number of Fixations**	GLOBAL ANALYSIS				
	PRESENTATION	61.30	.001	.54	Sim < Seq
	MODIFICATION	9.91	.00 3	16	Viol > No Viol
	EXPERTISE X PRESENTATION	12.84	.001	.20	If Sim, E < NE (.001); If Seq, E = NE (ns); Sim < Seq for E (.001) & NE (.01)
	AOIs	109	.001	.68	Target > others AOIs (.001) If Target, Seq=Sim (ns) ; if others AOIs Seq > Sim for M1 (.001),
	AOIs X PRESENTATION	4.85	.001	.09	M2(.006), M3 (.001), M5 (.001), M6 (.001), M7 (.001), M8 (.003 ); Key & M4(ns)
	AOIs X PRESENTATION X EXPERTISE	4.71	.001	.09	If NE, Seq < Sim for Target vs others AOIs (.001); if E (ns)
	AOIs X MODIFICATION	7.3 6	.001	.1	If Target, Viol > No Viol (.001), if M4 Viol < No Viol (.001), if M5 Viol > No Viol (.044); Key, M1, M2, M3 , M6, M7, M8 (ns)
	LOCAL ANALYSIS				
	PRESENTATION	5.49	.024	.11	Sim < Seq
	EXPERTISE X PRESENTATION	2 .78	.001	. 4	if E : Seq > Sim (.001); If NE : Sim> Seq (.068, ns)
	MODIFICATION	4.7	.0 5	.09	Viol > No Viol
	AOIs	162.42	.001	.78	Pre-Target > Post Target (.03 ); Target > pre-Target (.001); Target > post-Target (.001)
	AOIs X PRESENTATION	5.86	.004	.11	If pre-Target Seq > Sim (.027); if Target seq = Sim (ns); if Post-Target Seq > Sim (.001) If E, if pre-Target, Seq = Sim (ns) if Target seq > sim (.056);
	AOIs X PRESENTATION X EXPERTISE	7.18	.001	.14	if post-Target Seq > sim (.001) If NE if pre-Target seq = sim (ns); if Target Seq < sim (.001) if post-Target Seq = sim (ns)
	AOIs X MODIFICATION	25. 4	.001	. 6	If Pre-Target No Viol = Viol (ns); if Target Viol > No Viol (.001), If PostTarget No Viol > Viol (.00 )

Notes: ANOVA results for detection error rates, first fixation duration, dwell time, number of fixations at the global level (whole score = 10 AOIs) and the local level (pre-target, target and post-target measures of the score = 3 AOIs). Significant effects only are reported.
